# Systemic Amyloid Light-Chain (AL) Amyloidosis Revealed by Progressive Pan-Ungual Dystrophy: A Case Report

**DOI:** 10.7759/cureus.109691

**Published:** 2026-05-26

**Authors:** Meryeme Marhraoui, Syrine Hamada, Soukaina Haidouri, Mariame Meziane, Benzekri Laila

**Affiliations:** 1 Dermatology, Ibn Sina University Hospital Center, Mohamed V University, Rabat, MAR; 2 Hematology, Ibn Sina University Hospital Center, Rabat, MAR

**Keywords:** congo red staining, multiple myeloma, nail biopsy, nail dermoscopy, nail dystrophy, systemic amyloidosis

## Abstract

Systemic amyloid light-chain (AL) amyloidosis is a multisystem disorder caused by extracellular deposition of monoclonal immunoglobulin light chains. Cutaneous manifestations are diverse, but nail involvement is uncommon and may lead to diagnostic delay. We report the case of a 53-year-old man presenting with progressive dystrophy affecting all 20 nails for 18 months. Clinical examination revealed marked nail plate atrophy and onychorrhexis. Dermoscopy demonstrated nail plate atrophy, subungual hyperkeratosis, and splinter hemorrhages. A longitudinal lateral nail-bed biopsy revealed abundant amorphous eosinophilic deposits in the papillary dermis on hematoxylin and eosin staining. Congo red staining confirmed amyloid deposition. Subsequent systemic evaluation revealed markedly elevated free lambda light chains and bone marrow plasmacytosis, establishing the diagnosis of systemic AL amyloidosis associated with multiple myeloma. This case highlights that unexplained progressive pan-ungual dystrophy may represent an early manifestation of systemic amyloidosis. Early recognition and nail-unit biopsy can facilitate prompt diagnosis of this potentially fatal systemic disease.

## Introduction

Systemic amyloid light-chain (AL) amyloidosis is a multisystem disorder characterized by extracellular deposition of monoclonal immunoglobulin light-chain fibrils, leading to heterogeneous clinical manifestations, including mucocutaneous involvement [1]. Among the cutaneous manifestations of systemic amyloidosis, nail involvement is uncommon; however, progressive nail dystrophy may represent the initial and, in some cases, sole manifestation of systemic AL amyloidosis, particularly in the context of plasma cell dyscrasias [2,3]. Because nail changes in this setting are frequently misattributed to inflammatory nail disorders, such as nail lichen planus, diagnostic delay remains a significant concern [4]. We report a case in which progressive pan-ungual dystrophy represented the initial and predominant manifestation of systemic AL amyloidosis associated with multiple myeloma, as confirmed by nail-unit histopathology.

## Case presentation

A 53-year-old Moroccan man with no significant past medical history was referred to our dermatology department with an 18-month history of progressive, painless nail dystrophy affecting all 20 nails. Despite multiple prior consultations with various specialists, no diagnosis had been established, and the nail changes had remained unexplained. At the time of referral, the nail dystrophy represented the sole presenting complaint, with no overt systemic symptoms reported.

Clinical examination and dermoscopic findings

**Figure 1:**
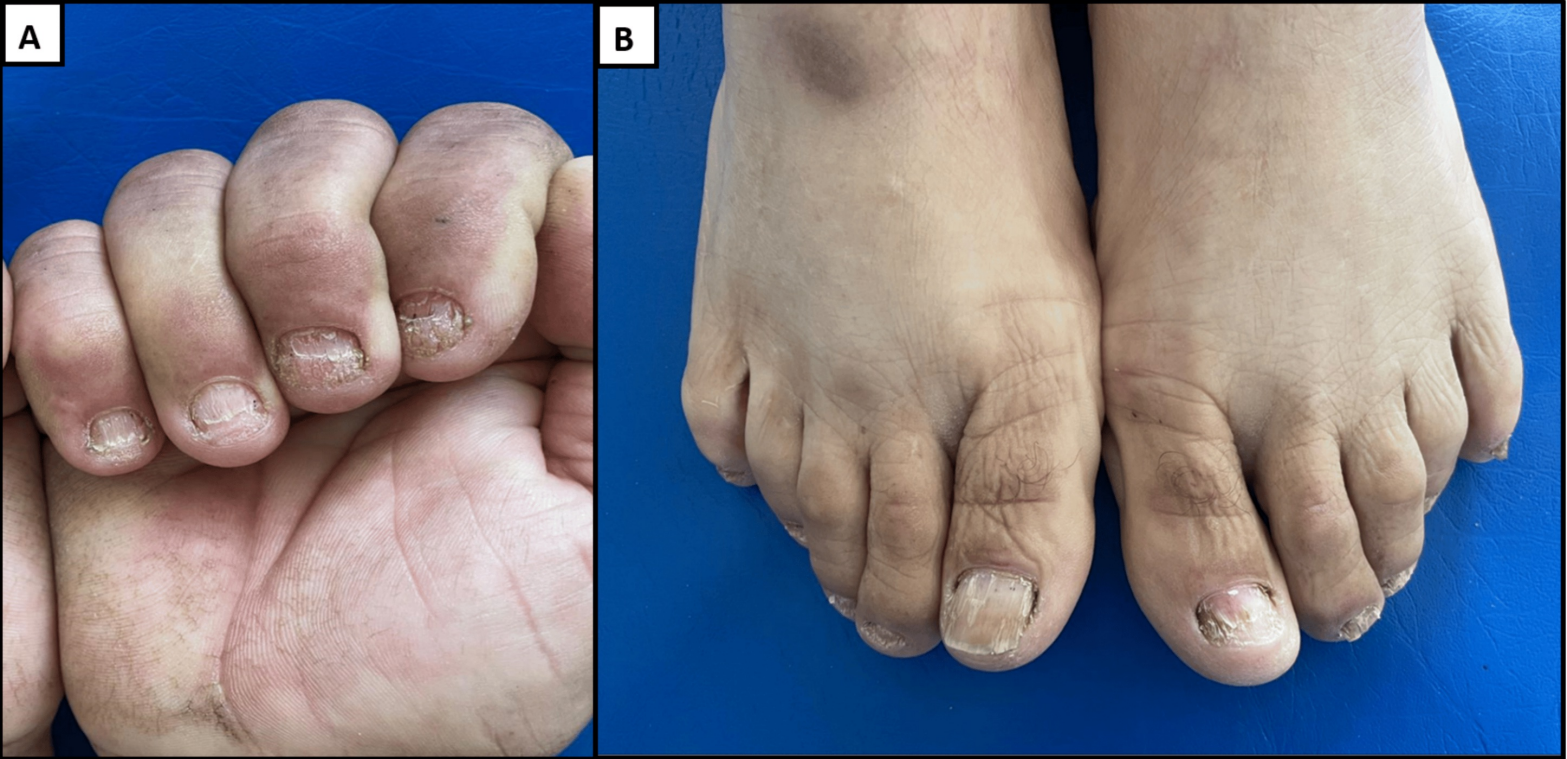
Clinical photographs showing diffuse nail dystrophy with nail plate atrophy and onychorrhexis involving the fingernails (A) and toenails (B).

Clinical examination revealed diffuse nail dystrophy affecting all fingernails and toenails (Figures 1A-1B), with nail plate atrophy and onychorrhexis. No lymphadenopathy or hepatosplenomegaly was noted.

**Figure 2:**
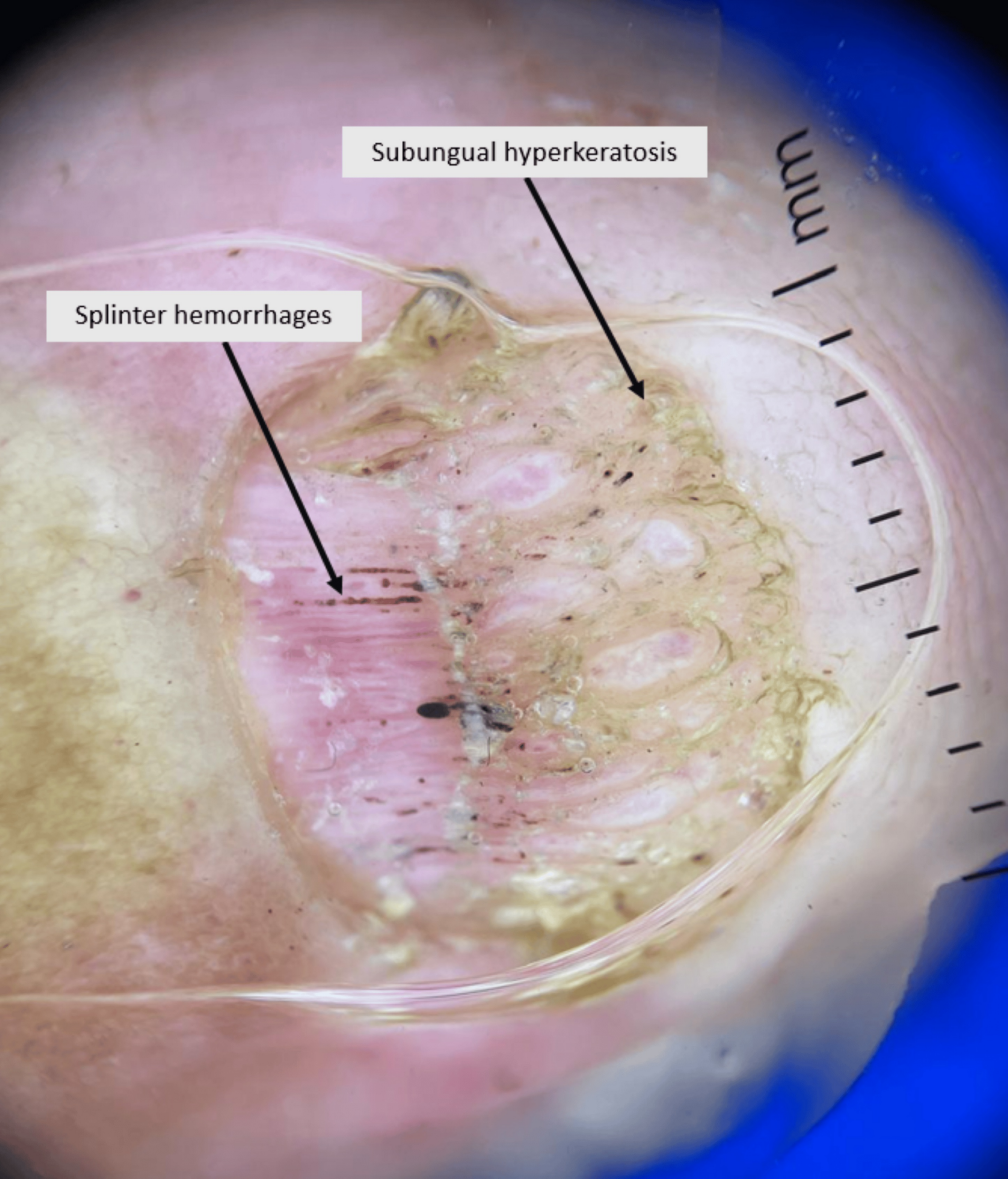
Nail-unit dermoscopy demonstrating nail plate atrophy, subungual hyperkeratosis, and splinter hemorrhages, using a polarized DermLite DL5 dermoscope with an ultrasound gel interface.

Nail dermoscopy demonstrated onychorrhexis, subungual hyperkeratosis, and splinter hemorrhages (Figure 2). Given the clinical suspicion of nail lichen planus or other inflammatory nail disorders, these findings prompted the decision to perform a nail-unit biopsy.

Histopathology and disease evolution

**Figure 3:**
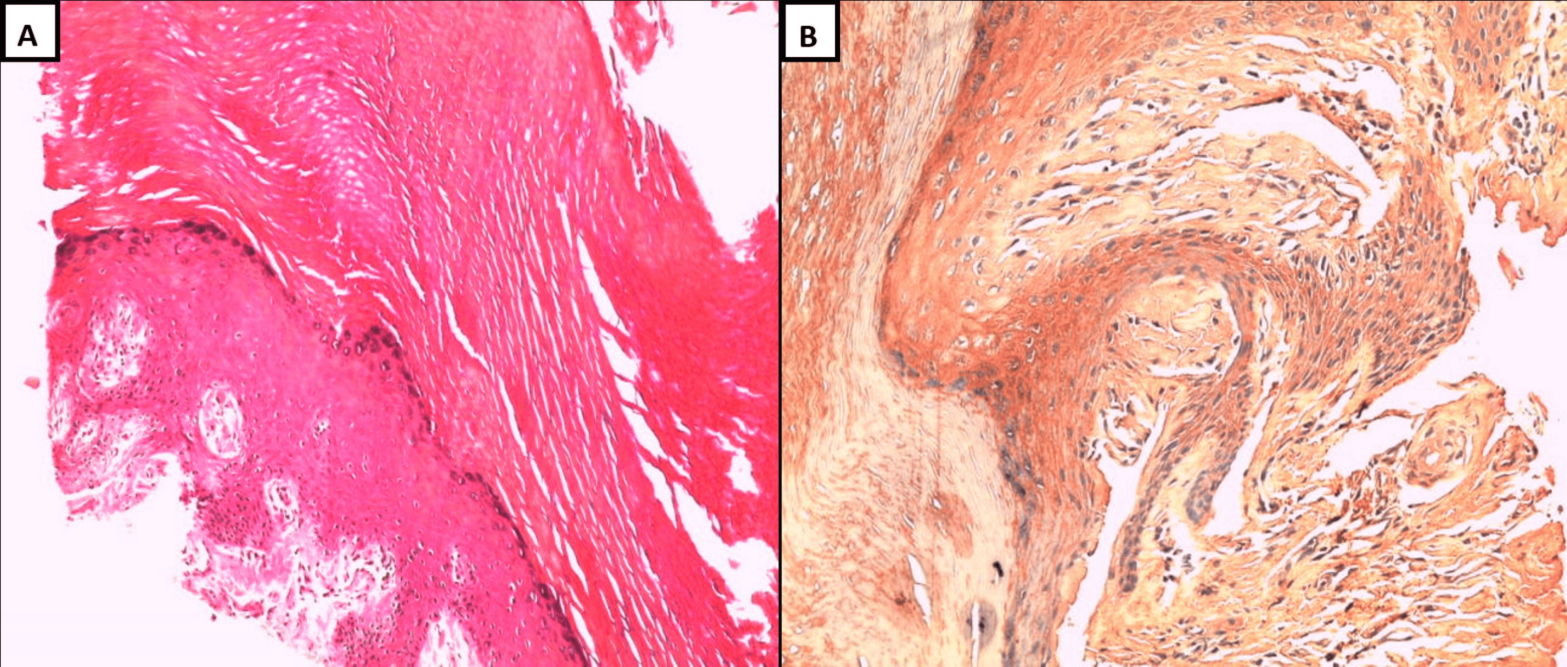
(A) Nail-bed histopathology showing abundant amorphous eosinophilic deposits within the papillary dermis on hematoxylin and eosin staining (H&E, ×100). (B) Congo red staining under nonpolarized light (×200) revealing extracellular amorphous orange-red deposits within the papillary dermis.

A longitudinal lateral nail-bed biopsy was performed. Hematoxylin and eosin staining revealed abundant amorphous eosinophilic deposits within the papillary dermis (Figure 3A). Congo red staining demonstrated extracellular amorphous orange-red deposits in the papillary dermis under nonpolarized light (Figure 3B). Apple-green birefringence under polarized light was observed by the dermatopathologist, establishing the diagnosis of amyloid deposition within the nail unit. 

**Figure 4:**
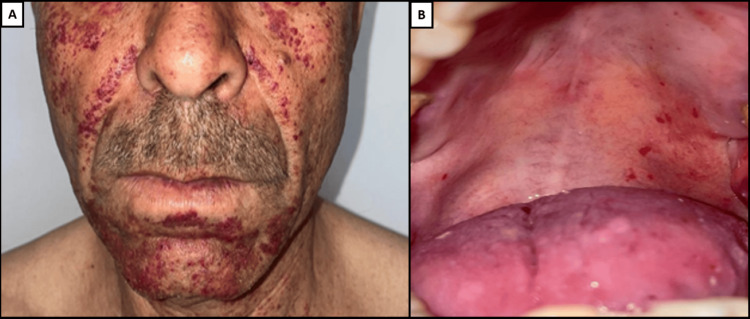
(A) Diffuse purpuric facial erythema. (B) Purpuric involvement of the oral mucosa and soft palate.

One month after the nail-unit biopsy, the patient developed new systemic manifestations, including purpuric facial erythema, periorbital purpura, and oral mucosal involvement (Figures 4A-4B), associated with exertional dyspnea and dysphonia.

The emergence of these findings strongly suggested an underlying evolving systemic process. Consequently, a complementary deep abdominal subcutaneous fat biopsy was performed, confirming systemic amyloidosis with Congo red-positive deposits, consistent with the nail-unit findings.

Systemic workup

Following histopathological confirmation of systemic amyloidosis, a comprehensive systemic evaluation was initiated. Serum protein electrophoresis revealed hypogammaglobulinemia. Serum-free lambda light chains were markedly elevated at 1,264.45 mg/L, and bone marrow examination demonstrated 39% plasma cell infiltration, collectively supporting the diagnosis of systemic light-chain (AL) amyloidosis associated with multiple myeloma.

Cardiac biomarkers were significantly elevated (high-sensitivity troponin T: 498 ng/L; NT-proBNP: 3,017.7 pg/mL). Echocardiography revealed left ventricular hypertrophy, diastolic dysfunction, and a reduced left ventricular ejection fraction of 37%, consistent with cardiac involvement in amyloidosis. Holter monitoring demonstrated sinus rhythm with frequent premature ventricular contractions (30/hour), nocturnal ventricular bigeminy, and monomorphic couplets, without atrial fibrillation or significant conduction abnormalities. The patient was classified as having Mayo 2012 stage III cardiac AL amyloidosis.

Renal function tests showed no evidence of renal involvement. Nasofibroscopy confirmed functional dysphonia in the absence of structural lesions. The complete details of the diagnostic workup are summarized in Table 1.

**Table 1:** Diagnostic workup of the patient. Abbreviations: hs-TnT, high-sensitivity troponin T; NT-proBNP, N-terminal pro-B-type natriuretic peptide; LVEF, left ventricular ejection fraction; AL, amyloid light chain

Category	Test Parameter	Result	Reference Range	Units
Protein Studies	Gamma globulins	3.69 - Markedly reduced (hypogammaglobulinemia)	8.00-13.50	g/L
Light Chain Analysis	Serum-free κ light chains	12.86	3.3-19.4	mg/L
	Serum-free λ light chains	1264.45 - Markedly elevated	5.71-26.30	mg/L
	κ/λ ratio	0.01 - Significantly decreased	0.26-1.65	Ratio
Bone Marrow Examination	Plasma cell infiltration	39% - Consistent with multiple myeloma	<5%	%
Cardiac Biomarkers	High-sensitivity troponin T (hs-TnT)	498 - Markedly elevated	<14	ng/L
	NT-proBNP	3017.7 - Markedly elevated	<125	pg/mL
Echocardiography	Left ventricular ejection fraction (LVEF)	37% - Reduced	55-70%	%
	Left ventricular hypertrophy	Present	Absent	Qualitative
	Diastolic dysfunction	Present	Absent	Qualitative
Holter Monitoring	Cardiac rhythm abnormalities	Frequent premature ventricular contractions (30/hour), nocturnal ventricular bigeminy, and monomorphic couplets	Absent	Qualitative
Renal Function	Creatinine	Normal	7-14	mg/L
	Urea	Normal	0.15-0.45	g/L
Nasofibroscopy	Laryngeal evaluation	Functional dysphonia without structural lesion	Normal	Qualitative
Histopathology	Nail-unit biopsy	Congo red-positive amyloid deposits with apple-green birefringence	Negative	Polarized light microscopy
	Abdominal fat pad biopsy	Congo red-positive amyloid deposits with apple-green birefringence	Negative	Polarized light microscopy
AL Amyloidosis Staging	Mayo 2012 cardiac staging	Stage III cardiac AL amyloidosis	Stages I-IIIb	Based on NT-proBNP and hs-TnT thresholds

Treatment and outcome

Treatment was initiated with bortezomib-based therapy in combination with a sodium-glucose cotransporter 2 (SGLT2) inhibitor for heart failure management. Despite these interventions, the patient's condition deteriorated rapidly, and he died from complications related to advanced cardiac amyloidosis before cardiac magnetic resonance imaging could be performed.

## Discussion

This case highlights progressive pan-ungual dystrophy as a potentially underrecognized manifestation of systemic AL amyloidosis, particularly in patients with multiple myeloma, in whom nail involvement may precede other cutaneous or systemic manifestations by months to years [2-4].

Systemic amyloidosis is rarely suspected in patients presenting with progressive nail dystrophy, as these abnormalities are more commonly attributed to inflammatory, infectious, or traumatic nail disorders. Nail lichen planus represents the principal diagnostic consideration because it may manifest with longitudinal ridging, fissuring, nail thinning, trachyonychia, and dorsal pterygium [4,5]. However, histopathological examination in nail lichen planus typically demonstrates a lichenoid interface dermatitis, which was absent in the present case. Onychomycosis was excluded by negative periodic acid-Schiff staining of the nail-unit biopsy specimen, while psoriatic nail disease was not supported by the clinical or dermoscopic findings [6]. Traumatic or idiopathic 20-nail dystrophy was also considered unlikely given the patient's age and overall clinical context. Because nail involvement is rarely the initial manifestation of systemic amyloidosis, the diagnosis is frequently overlooked at presentation, contributing to substantial diagnostic delay.

Several reports have illustrated this diagnostic challenge. Breathnach et al. described a landmark case in which nail abnormalities, including longitudinal striation, crumbling, and brittleness, preceded other cutaneous manifestations by five months and ultimately led to biopsy confirmation of amyloid deposition [2]. Similarly, Fanti et al. reported severe onychodystrophy affecting all digits that was initially misdiagnosed as nail lichen planus for three years before histopathological confirmation of primary systemic amyloidosis [3]. More recently, Oberlin et al. documented diffuse onychodystrophy involving all 20 nails in a patient with multiple myeloma-associated AL amyloidosis, further emphasizing the marked clinical overlap with inflammatory nail disorders [4]. In the present case, an 18-month diagnostic delay occurred despite extensive pan-ungual involvement, thereby underscoring the importance of heightened clinical awareness.

The spectrum of nail abnormalities reported in systemic amyloidosis is broad and includes longitudinal ridging, nail plate atrophy, onychorrhexis, splinter hemorrhages, subungual hyperkeratosis, onycholysis, and anonychia [2-8]. In our patient, the dermoscopic combination of onychorrhexis, subungual hyperkeratosis, and splinter hemorrhages represented an important diagnostic clue and directly guided the decision to perform a nail-unit biopsy. In this context, nail-unit dermoscopy may serve as a valuable noninvasive adjunct in the evaluation of unexplained multi-nail dystrophy, particularly when an underlying systemic disorder is suspected [5,6].

Histopathological confirmation using Congo red staining demonstrating apple-green birefringence under polarized light remains the diagnostic gold standard for amyloidosis [1-5]. In the present case, the nail-bed biopsy proved pivotal not only in establishing the diagnosis but also in prompting a targeted systemic evaluation, which revealed markedly elevated serum free lambda light chains and bone marrow plasmacytosis, thereby confirming AL amyloidosis associated with multiple myeloma. These findings support maintaining a low threshold for nail-unit biopsy in patients presenting with progressive multi-nail dystrophy of unclear etiology, particularly in the presence of systemic symptoms, atypical clinical features, or treatment resistance. Pathophysiologically, amyloid deposition within the nail matrix and nail bed, with preferential involvement of the proximal matrix, likely underlies the observed nail fragility, longitudinal ridging, and dystrophic changes [4].

The association between nail involvement and plasma cell dyscrasias is well established [2,3,8]. As emphasized by Renker et al., early recognition of nail manifestations is essential for timely identification of the underlying hematologic disorder and initiation of appropriate management [9]. Clinicians should therefore maintain a high index of suspicion for systemic amyloidosis in patients presenting with unexplained progressive multi-nail dystrophy, particularly when systemic manifestations are present, or treatment resistance is observed.

Finally, distinguishing systemic amyloidosis with nail involvement from localized nail-unit amyloid deposition (nail amyloidoma) is critical because these entities differ substantially in both prognosis and management [10]. Unlike localized amyloidoma, systemic AL amyloidosis requires comprehensive hematologic evaluation and multidisciplinary management. Awareness of this distinction may therefore facilitate timely diagnosis, reduce diagnostic delay, and improve patient outcomes.

## Conclusions

Progressive dystrophy involving multiple nails should prompt consideration of systemic amyloidosis. Nail-unit biopsy with Congo red staining can provide an early and definitive diagnosis, enabling timely systemic evaluation and management. Dermatologists play a pivotal role in recognizing this rare yet clinically significant presentation of a potentially life-threatening disease.

## References

[1] Panagopoulos F, Jahaj E, Papaodyssea I, Stamatopoulos V, Kounatidis D, Vallianou N (2024). Cutaneous amyloidosis in a patient with systemic amyloidosis due to multiple myeloma. Health Res J.

[2] Breathnach SM, Wilkinson JD, Black MM (1979). Systemic amyloidosis with an underlying lymphoproliferative disorder. Report of a case in which nail involvement was a presenting feature. Clin Exp Dermatol.

[3] Fanti PA, Tosti A, Morelli R, Galbiati G (1991). Nail changes as the first sign of systemic amyloidosis. Dermatologica.

[4] Oberlin KE, Wei EX, Cho-Vega JH, Tosti A (2016). Nail changes of systemic amyloidosis after bone-marrow transplantation in a patient with multiple myeloma. JAMA Dermatol.

[5] Litaiem N, Chabchoub I, Gara S, Slouma M, Hamdi MS, Zeglaoui F (2021). Nail changes in systemic amyloidosis. Clin Case Rep.

[6] Dehavay F, Richert B (2021). Nail is systemic disorders: main signs and clues. Dermatol Clin.

[7] Mancuso G, Fanti PA, Berdondini RM (1997). Nail changes as the only skin abnormality in myeloma-associated systemic amyloidosis. Br J Dermatol.

[8] Fujita Y, Tsuji-Abe Y, Sato-Matsumura KC, Akiyama M, Shimizu H (2006). Nail dystrophy and blisters as sole manifestations in myeloma-associated amyloidosis. J Am Acad Dermatol.

[9] Renker T, Haneke E, Röcken C, Borradori L (2014). Systemic light-chain amyloidosis revealed by progressive nail involvement, diffuse alopecia and sicca syndrome: report of an unusual case with a review of the literature. Dermatology.

[10] Bonito F, Kolivras A, Sass U, Richert B (2023). Nail amyloidoma: two case reports of a new entity. Skin Appendage Disord.

